# Predicting Functional Diversity Within Enzyme Families: A Practical Perspective

**DOI:** 10.1002/cbic.70568

**Published:** 2026-10-09

**Authors:** Nika Sokolova, Kristina Haslinger

**Affiliations:** ^1^ Department of Health Sciences and Technology ETH Zürich Zürich Switzerland; ^2^ Department of Chemical and Pharmaceutical Biology University of Groningen Groningen The Netherlands

**Keywords:** biocatalysis, enzymes, high‐throughput screening, machine learning, substrate scope

## Abstract

Machine learning has become an increasingly powerful tool in enzyme discovery, enabling the prediction of enzyme activity, substrate scope, and functional specificity across diverse protein families. However, many reported models rely on narrowly sampled datasets and task‐specific benchmarks, limiting their robustness and generalizability to distantly related enzymes or novel chemical substrates. In this concept article, we provide a practical, best‐practice–oriented perspective on developing enzyme family‐ or class‐specific machine learning (ML) predictors with an emphasis on data quality, representation, and model interpretability. We first discuss strategies for assembling biochemical datasets from literature, highlighting common sources of bias, redundancy, and uncertainty impacting downstream model performance. We then outline key considerations for generating new experimental data that are better suited for ML applications, including dataset design and coverage of enzyme and chemical space. Next, we summarize core computational steps involved in constructing enzyme and substrate representations, training and testing predictive models, and analyzing learned embedding spaces. Finally, we discuss outstanding technical challenges, such as mitigating taxonomic sampling bias, computing representations of enzymes with cofactors and post‐translational modifications, and integrating predictions into design–build–test–learn cycles for real‐world applications.

## Graphical abstract

Integrating machine learning into enzyme discovery requires navigating the trade‐offs between data quantity and quality. This article provides a best‐practice framework for developing robust predictors, focusing on mitigating bias in biochemical datasets, implementing systematic “cold‐drug” validation strategies, and utilizing explainable artificial intelligence to translate black‐box predictions into actionable biocatalytic insights.

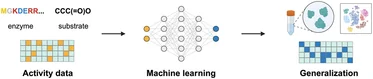

## 1 Introduction

With the ever‐accelerating development of next‐generation sequencing technologies, the number of sequences in genomic and protein databases continues to rise. Paradoxically, while the community is gaining access to more of these “biochemistry blueprints,” the vast majority of the sequences in these databases remain functionally uncharacterized [1]. Recent advances in functional categorization heavily rely on machine learning algorithms to predict gene ontology (GO)—a structured, standardized vocabulary that defines biological processes, cellular components, and molecular functions—and enzyme commission (EC) classification [2, 3]. These rather broad classifications can be extremely valuable for predicting the metabolic capability of previously uncharacterized organisms or entire microbial communities [4, 5] or predicting the function of genes previously annotated as encoding “domains of unknown functions” (DUFs) [6, 7]. However, they do not provide sufficient resolution to identify the exact biological function of enzymes in the context of natural biosynthetic pathways [8] or their potential for being repurposed in engineered biosynthetic pathways or biocatalytic cascades [9].

For such prediction tasks, specific machine learning models are necessary that can predict the interaction of enzymes with their potential substrates. While these algorithms could be trained on detailed experimental data such as binding affinities and/or enthalpies [10, 11], enzyme kinetics (*k*_cat_) [11, 12, 13, 14], and structural information [15, 16], the majority of models are currently trained on binned substrate conversion data. Herein, general prediction models are not specific for certain enzyme superfamilies or classes and have been shown to perform well for enzymes and substrates similar to the training set [10, 17, 18, 19]. However, their performance drops sharply for prediction tasks for enzymes sharing less than 40 % sequence identity with characterized enzymes and for previously untested substrates [17]. Therefore, several enzyme‐family‐specific machine learning algorithms have been trained that aim to fill this gap and provide reliable predictions for real‐world biocatalytic applications. Many of these tools have been converted into user‐friendly protocols or servers to ensure broad usability [20, 21, 22, 23, 24, 25]. Beyond academic interest, these approaches are also receiving increasing attention in industry, as exemplified by IBM’s RoboRXN tool that matches specific catalytic steps with biological catalysts [26].

Despite the utility of these emerging applications, recent comparative studies have analyzed the generalizability of certain models across various prediction tasks and have found that the nature of the underlying training data set and the algorithms used to generate the input representations have a strong influence on the generalizability of the trained predictor [27, 28, 29]. Thus, this concept article aims to provide a best‐practice guide for researchers interested in training such enzyme family‐ or class‐specific machine learning predictors with high predictive accuracy and broad generalizability toward distantly related enzymes and novel substrates of interest (Figure 1). We first discuss the strategies for gathering the biochemical input data from literature and the caveats of this approach, followed by important considerations when generating new biochemical data for machine learning. Subsequently, we provide a brief overview of the computational steps for generating enzyme and substrate embeddings and training the algorithms. Along the way, we point out excellent method reviews as well as comprehensive reviews on the latest tools and use cases [8, 9, 28, 30]. In the perspective section, we discuss several technical challenges that still need to be addressed by the field.

**FIGURE 1:**
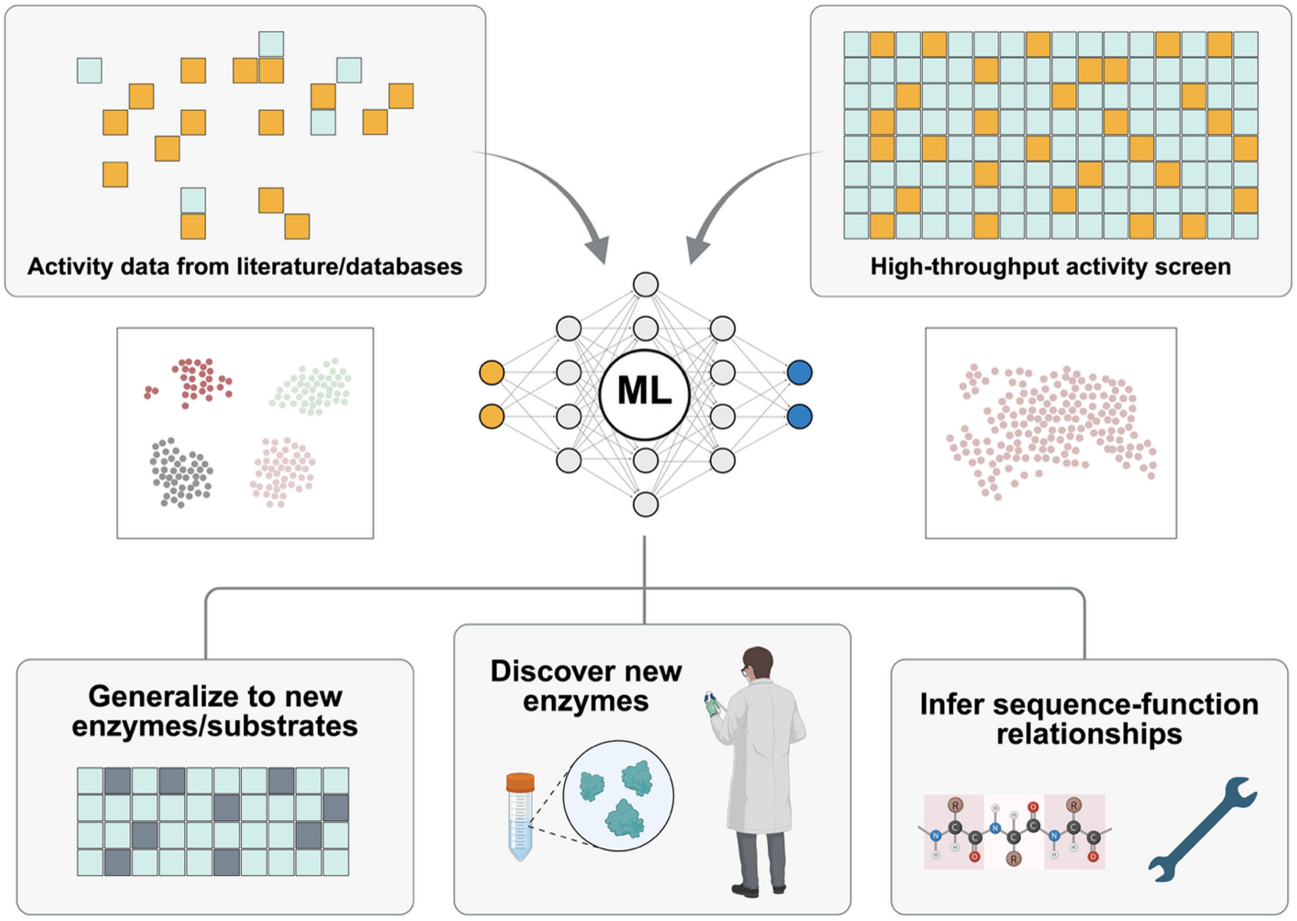
Predicting functional diversity within enzyme families with machine learning (ML) from literature data or in‐house data. Created in BioRender. Sokolova, N. (2026). https://BioRender.com/desnygp.

## 2 Leveraging Existing Experimental Data

Historically, some protein families have been characterized better than others, and published experimental data can be leveraged to infer sequence–function relationships or predict substrate specificity by training ML algorithms. These data can be manually curated from literature, or they can be retrieved from protein databases such as BRENDA [31], UniProt [32], EnzyMine [33], and others [34]. The vast majority of currently available ML models for enzyme‐substrate specificity prediction were trained on such literature/database data (Table 1). For instance, protein kinases, catalyzing the phosphorylation of serine, threonine, histidine, or tyrosine residues, were among the first enzyme families to benefit from ML‐assisted substrate specificity prediction. At least five different ML models for phosphorylation site prediction [46, 47, 48, 49, 50] were trained on data from public databases like Phospho.ELM [57], PhosphoSitePlus [58], and PhosphoNetworks [59]. More recently, Zhou et al. used the exceptionally comprehensive dataset on substrate specificity for human protein kinases [60], which included both positive and negative data, showing an improvement in zero‐shot learning [24]. In their deep evolutionary analysis of the glycosyltransferase A (GT‐A) family, Taujale et al. [44] mined 713 GT‐A sequences with known donor sugars from CAZy [61] and Uniprot [32] databases and used the data to train a machine learning model for GT‐A substrate specificity prediction. Numerous ML tools have been developed to predict substrate specificity of assembly‐line enzymes from natural product biosynthetic pathways, namely, type I polyketide synthases [37] and nonribosomal peptide synthetases [21, 22, 40, 41, 42, 51]. In several cases, these approaches led to the discovery of novel biosynthetic enzymes or entire biosynthetic gene clusters [37, 40, 41, 42]. Many other enzyme families have extensive experimentally validated data in public repositories and the literature [62, 63, 64, 65, 66, 67, 68, 69, 70, 71, 72, 73, 74], representing a resource for future machine learning‐based mapping of sequence–function relationships.

**TABLE 1:** Family‐specific and general machine learning classifiers for the prediction of enzyme–substrate specificity. This list is not exhaustive. ACP—acyl carrier protein, PET—polyethylene terephthalate, BGC—biosynthetic gene cluster, and NRPS—nonribosomal peptide synthetase.

Classifier type	Name/enzyme class	Experimental validation/application	Reference
**Based on literature data or database entries**			
General	EZSpecificity	Validated on 8 new enzymes and 78 substrates	[19]
General	CLEAN	Validated on 36 new enzymes	[35]
Family‐specific	Acyl‐ACP thioesterase	Identified two new enzymes	[36]
Family‐specific	Type I polyketide synthases	Identified a new BGC	[37]
Family‐specific	PET hydrolases	Identified 37 new thermotolerant enzymes	[38]
Family‐specific	Glycosyltransferases	Validated on 1001 new enzyme–substrate pairs	[39]
Family‐specific	NRPS–DeepAden	Identified two new BGCs	[40]
Family‐specific	NRPS–PARAS	Identified a new BGC	[41]
Family‐specific	NRPS–NRPStransformer	Identified novel antimicrobial peptides	[42]
Family‐specific	Multicopper oxidases	Identified 8 new enzymes with improved catalytic properties (specific activity, thermal stability, pH range, organic solvent tolerance)	[43]
Family‐specific	L‐asparaginases	Identified 4 new enzymes with higher specific activity	[43]
Family‐specific	Glycosyltransferases A	No	[44]
Family‐specific	Glycosyltransferase B	No	[45]
Family‐specific	Kinase–MusiteDeep	No	[46]
Family‐specific	Kinase–PhosIDN	No	[47]
Family‐specific	Kinase–Phosformer	No	[48]
Family‐specific	Kinase–Phosformer‐ST	No	[24]
Family‐specific	Kinase–DeepPhos	No	[49]
Family‐specific	Kinase–EMBER	No	[50]
Family‐specific	NRPS–NRPSpredictor2	No	[22]
Family‐specific	NRPS–AdenPredictor	No	[51]
Family‐specific	NRPS–SANDPUMA	No	[21]
Family‐specific	Hydrolases	No	[52]
General	ESP	No	[17]
General	ProSmith	No	[10]
General	FusionESP	No	[18]
General	EasIFA	No	[16]
**Based on a dedicated dataset generated in‐house**			
Family‐specific	Glycosyltransferases 1	Inferred and validated sequence–function relationships	[53]
Family‐specific	Fe(II)/α‐ketoglutarate‐dependent oxygenases	Validated on one enzyme and 3 new substrates	[54]
Family‐specific	Type III polyketide synthases	Validated on 5 new enzymes and 12 new substrates	[55]
Family‐specific	Thiolases	No	[25]
Family‐specific	Nitrilases	No	[56]

Beyond curated database queries, recent developments in natural language processing (NLP) and large language models (LLMs) have substantially accelerated the automated extraction of enzyme–substrate relationships directly from literature [75, 79]. Advanced LLM‐driven text‐mining workflows and named entity recognition pipelines can now extract kinetic parameters, substrate specificities, and reaction schemes at scale with increasing accuracy. While these automated literature extraction methods offer a scalable avenue to generate high‐density training data, careful downstream validation remains essential to filter out extraction errors and hallucinated relations [80].

While existing datasets have proven useful for training ML models, functional annotations in public databases often rely on automated sequence‐homology‐based transfers that carry propagated errors or lack experimental validation [81]. Existing datasets may also exhibit variability in the conditions for experimental testing (pH, temperature, buffer composition, cosolvent concentration, protein purity) and positivity bias due to the chronic underreporting of negative results [82]. As a result, models risk learning database‐specific noise or environmental artifacts rather than fundamental rules of enzyme–substrate interactions, severely diminishing model generalizability when applied to real‐world biocatalytic conditions. While there are computational strategies to mitigate this (see Section 4), literature data often cannot fully substitute for high‐quality, internally generated data. Therefore, collecting “dense” experimental data in‐house is essential for activity profiling of enzyme families with few or even no characterized members.

## 3 Generation of New Experimental Data for Machine Learning

Successful machine learning begins in the wet lab, long before the first algorithm is trained. Too often, datasets are generated with a narrow focus—sampling of only a few closely related sequence clades or a handful of chemically similar substrates—which severely limits the model’s ability to generalize to novel biochemical space. To mitigate this coverage bias, several studies have moved beyond traditional one‐to‐many screening strategies in favor of a “matrix‐like” approach, where a diverse set of homologous enzymes is tested against an expansive library of chemical scaffolds. This high‐density data generation allows for the training of algorithms that can accurately extrapolate predictions across the “natural” sequence space of an enzyme family.

### 3.1 Selection of Enzymes

The pool of enzyme candidates can be retrieved from protein databases like UniProt [32] and InterPro [83] through a sequence homology search or from enzyme databases such as BRENDA [31] using the EC number. To narrow down the search space, taxon‐specific genomic databases can also be queried (e.g., MycoCosm [84] for fungi, Phytozome for plants [85], or P10K database for protists [86]). Using targeted phylogenetic approaches, especially with underexplored taxa like fungi and archaea, may yield enzyme homologs that possess superior stability or unique substrate specificity based on their evolutionary origin [87, 88]. Furthermore, multimodal frameworks such as ProTrek allow researchers to perform enzyme searches using natural language input, sequence, or structure information, bridging textual activity descriptions with sequence space without requiring homology alignments [89].

When selecting candidate enzymes for experimental testing, the goal is to maximize diversity while minimizing redundancy, ensuring that the experimental data provide the most information about the family’s functional landscape. Hence, the sequence space typically undergoes some form of clustering, with the most employed approaches being phylogenetic trees and sequence similarity networks (SSNs) [90]. Phylogenetic trees are built from multiple sequence alignments and use statistical models to infer the most likely path of evolution from a common ancestor. SSNs, on the other hand, employ clustering based on pairwise sequence identity and are particularly useful at scale, enabling clustering of thousands of sequences. Candidates are then selected from each phylogenetic clade or SSN cluster using normalized mean pairwise similarity or network centrality to identify centrally positioned (representative) sequences [91], while additional candidates may be chosen to capture taxonomic, motif, or active‐site diversity. Positional Shannon entropy calculated from a multiple sequence alignment can be used independently to quantify residue variability across the selected set [64]. Under a base‐2, 20‐amino‐acid formulation, entropy ranges from 0 bits for a fully conserved alignment position to 4.32 bits for maximum positional diversity, but it is also often analyzed as a normalized value ranging from 0 to 1 [92].

With the integration of AlphaFold2‐predicted structures [93] in the UniProt database, clustering by structure rather than sequence is also possible, and dedicated methods have been developed for structural similarity filtering [94]. An emerging alternative involves using protein language models to generate high‐dimensional numerical embeddings [95]. By clustering these embeddings, researchers can capture complex contextual and structural information directly from sequence data, allowing for the identification of functional “islands” and sampling blind spots that are often invisible to traditional identity‐based or phylogenetic methods [70].

### 3.2 Selection of Substrates

The choice of substrates for activity screening is typically based on the literature precedent and is highly dependent on the commercial and/or synthetic accessibility of the chemicals in question. However, this approach often creates a “sparse” matrix that lacks the diversity required for robust machine learning, limiting the predictive power the results will have [96, 97]. Furthermore, overly relying on “dense‐but‐narrow” chemical panels—characterized by small structural variations, such as the shifting of a single methyl or hydroxyl group—can lead to models that exhibit high internal accuracy but fail to generalize beyond a specific scaffold, a problem known as sampling bias [96]. To prevent that, simple descriptors such as logP (partition coefficient, a measure of lipophilicity) are commonly used to assess chemical variability in the substrate set [98]. More sophisticated methods of chemical space analysis are increasingly employed to ensure that the chosen substrate panel is representative of the desired reaction space. For instance, encodings like ECFP4/ECFP6 (extended‐connectivity fingerprints) or MACCS (Molecular ACCess System) keys translate molecular connectivity into bit vectors, allowing for the calculation of Tanimoto similarity scores [25] or more elaborate metrics such as the myopic maximum common edge subgraph distances [96]. This enables the rational selection of a substrate library that maximizes structural coverage while minimizing experimental redundancy.

### 3.3 Gene Expression and Activity Screening

Heterologous protein expression in *Escherichia coli* followed by activity testing with purified enzymes or cell‐free lysates remains the conventional strategy for characterization of candidate enzymes. However, the requirement for molecular cloning, transformation, and cell lysis creates a substantial experimental bottleneck. As the search space expands to hundreds of enzymes and thousands of potential reactions, more streamlined platforms are required. Cell‐free expression (CFE) has emerged as a high‐throughput alternative, offering a “plug‐and‐play” framework that bypasses the weeks‐long cycles of traditional microbial cultivation [99]. By eliminating the need for clearing lysates and managing cell viability, CFE allows for the rapid transition from digital sequence to functional assays. This approach has been used to map the substrate specificity landscapes of *N*‐glycosyltransferases [100], *O*‐methyltransferases, type III polyketide synthases, and amide synthetases [55, 101, 102]. The utility of CFE platforms is expected to rise further as the commercial landscape diversifies beyond traditional *E. coli* lysates [103, 104] into eukaryotic systems [105, 106].

The analytical part of the workflow—detecting and quantifying product formation—requires equally scalable technologies to generate the dense matrices needed for ML. However, throughput must be balanced with feasibility: while qualitative readouts (active/inactive) are sufficient for binary classifiers, quantitative data (yield, *K*_cat_, *K*_m_) is essential for regressive models. Traditional liquid chromatography coupled to mass spectrometry remains the gold standard for accuracy but is often too slow for libraries comprising thousands of enzyme/substrate combinations. Recent advances in mass spectrometry approaches, as reviewed by Shepherd et al., have substantially reduced analysis times [107], enabling, for example, the screening of approximately 20,000 halogenase reactions in a single study [63]. As an alternative, microfluidic screening platforms are emerging as powerful tools for scalable enzymatic profiling [108]. In particular, droplet‐based microfluidics has recently been used to profile substrate specificity, catalytic efficiency, and thermostability of 24 haloalkane dehalogenases, illustrating its potential for generating rich, multidimensional datasets at high throughput [65].

## 4 Training and Testing of Machine Learning (ML) Algorithms

Before starting the training, it is important to first assess the quality of the training data set collected from literature or in‐house data: size of the dataset, balance of true positives and true negatives, sequence, and chemo‐diversity. For binary classification of specific reaction or substrate types within a single enzyme family, models typically require 10^2^ to 10^3^ of high‐quality experimental data points. Broad generalist models or regression tasks predicting kinetic parameters generally demand more data points (at least 10^4^ to 10^5^) to achieve robust generalization across diverse sequence space. In particular, for smaller datasets, a good balance between positive and negative data points is crucial and cannot be easily corrected or compensated for. In the case of positivity bias in large, typically literature‐derived datasets, a common correction strategy is introducing synthetic negative data points through random sampling [17]. If, however, negative data points dominate, it is possible to assign a lower weight to the overrepresented negative data points during training to account for the imbalanced training dataset, as employed in ProSmith [10]. Finally, sequence and chemodiversity can be assessed by performing principal component analysis (PCA), t‐distributed stochastic neighbor embedding (t‐SNE), or uniform manifold approximation and projection (UMAP) analysis of the machine‐readable, molecular representations of the input sequences and substrates. For large datasets, dereplicating or rebalancing by removing data points from overrepresented sequence clades or groups of substrates may be possible.

Once the appropriateness of the data set is established, a training and validation strategy needs to be chosen. Proper train–test splitting is essential to obtain unbiased performance estimates and to avoid overly optimistic results. To prevent the model from simply “memorizing” chemical motifs, it is essential to move beyond random data splitting. Several classifiers [10, 49] emphasize the use of cold‐drug splitting, a concept borrowed from the broader field of drug–target interactions: ensuring that none of the substrates in the test set appear anywhere in the training set. The same can be achieved for test enzymes by excluding entire clades/clusters from the training set so that the test set has low sequence similarity to the training set. A cut‐off of 60% is often used but will overestimate the accuracy of the trained model; thus, 40% is the recommended threshold [28].

Next, the enzymes and substrates need to be encoded into numerical representations for processing by machine learning algorithms. A detailed review on creating protein representations was recently published by Harding–Larsen et al., highlighting that current trend is to use learned embeddings generated with pretrained algorithms such as ESM2 and ProtT5 for this task [95]. However, for specific enzyme families or sequence spaces with low homology (<40% identity) to known query proteins, generic embeddings may fail to distinguish subtle functional nuances. To overcome this, it is possible to fine‐tune the core neural network on targeted, experimentally validated family datasets—adjusting the model’s internal parameters rather than keeping them fixed. This task‐specific fine‐tuning reshapes the latent embedding space so that functionally equivalent enzymes cluster together despite low sequence identity, significantly enhancing predictive accuracy and generalizability for remote homologs [109]. User‐friendly platforms like SaprotHub simplify this process, enabling noncoders to deploy and fine‐tune structure‐aware language models without complex programming [110]. To create representations of chemical entities, there are also multiple methods that are widely used in cheminformatics [111, 112]. In the context of enzymology, however, there is no clear trend as to which method is better. Weighing the computing cost of more sophisticated representations against the actual benefits will lead to different conclusions depending on the machine learning task at hand.

Regardless of the chosen method to create the numerical representations, they can be generated separately and then concatenated as input for training of classic, standalone classifiers, including support vector machines (SVM), k‐nearest neighbor (k‐NN) models, decision tree‐based models, gradient boosting machines (GBM), and multilayer perceptrons (MLPs). However, recent approaches have found success in simultaneously processing enzyme sequences and substrate strings in the same input [10]. These methodologies span four primary neural paradigms: graph neural networks (GNNs), transformer‐based protein language models (pLMs), geometric deep learning, and multimodal crossattention networks, each utilizing distinct architectural mechanisms to capture structural, sequence, or spatial relationships (Table 2). For an in‐depth breakdown of individual strategies and selection criteria, readers are directed to comprehensive methodological reviews [113, 114].

**TABLE 2:** Key machine learning architectures for enzyme–substrate interaction modeling.

Architecture	Core input representation	Key architectural mechanism	Primary advantage for biocatalysis
**Graph neural networks (GNNs)**	2D Molecular graphs (atoms and bonds)	Iterative message‐passing across atomic neighborhood graphs	Learns structural features without fixed fingerprint schemas
**Transformers/pLMs**	FASTA sequences and SMILES strings	Multihead self‐attention over token sequences	Captures long‐range evolutionary signatures and global sequence context
**Geometric deep learning**	3D atomic coordinates (*x, y, z*)	SE(3)‐equivariant vector/graph convolution	Encodes active‐site spatial geometry and stereochemical constraints
**Multimodal/crossattention**	Dual input (e.g., pLM embeddings + 2D/3D graphs)	Crossattention fusion into joint latent space	Dynamically models residue–substrate pair interactions and binding fit

In the case of end‐to‐end GNN or transformer architectures, where the input embeddings are dynamically adapted during the learning process, it is possible to analyze the task‐conditioned embeddings after training. This so‐called latent‐space analysis can provide insight into the learning process and whether true biochemical information was learned rather than dataset‐specific correlations [115, 116, 117, 118, 119].

To further improve representation quality and counteract data scarcity or positivity bias, contrastive learning has emerged as a particularly powerful strategy. Rather than relying solely on conventional classification losses, frameworks such as CLEAN [35] use contrastive learning to evaluate distance relationships between positive and negative enzyme substrate pairs. By explicitly pulling functionally similar enzymes together in embedding space while pushing distinct functional classes apart, contrastive paradigms make representation learning far more sample‐efficient, maintaining high predictive performance even on sparse or imbalanced datasets.

After model training, it is evaluated using the previously excluded test set to determine its reliability. When assessing model performance, simple metrics like accuracy can be deceptive, especially with imbalanced datasets common in biocatalysis. For instance, in a dataset where 95% of enzyme–substrate pairs are inactive, a model can achieve 95% accuracy just by predicting “Inactive” every time. Thus, we recommend employing several different metrics, such as area under the receiver operating characteristic curve (ROC–AUC), Matthews correlation coefficient (MCC), and F1 score, to reveal unique aspects of the model’s performance and practical utility [120].

The ultimate utility of any classifier rests on its experimental validation, particularly its performance on enzymes, new substrates, or even new enzyme/substrate pairs. To rigorously assess generalizability, it is essential to test the model on enzymes with low homology to those in the training set or on substrates with significantly different chemical architectures [28]. Indeed, several studies have successfully leveraged ML to identify enzymes with unusual substrate specificities [19, 36, 41, 54, 55]. In some cases, the black‐box predictions can be translated into explainable insights that help infer sequence–function relationships within enzyme families. For instance, DeepAden helped identify a flexible binding pocket unique to cysteine‐specific A‐domains in NRPSs [40], while Phosformer‐ST proposed specificity‐determining residues in the peptide sequence [24]. GT‐Predict pinpointed several residues as the drivers of substrate recognition in superfamily 1 glycosyltransferases; site‐directed mutagenesis confirmed these predictions by shifting the specificity of mutant enzymes toward new substrates [53]. These examples highlight the potential of ML models for use in rational enzyme engineering and biocatalyst discovery.

## 5 Summary and Outlook

Machine learning has taken a central role in predicting compound–protein interactions across diverse fields, including medicinal chemistry, metabolic modeling, and biocatalyst mining [8, 9, 28, 121]. However, systematic benchmarking studies demonstrate that models appearing highly predictive under random train–test splits often fail when evaluated on novel substrates or distantly related enzymes [27, 29, 122]. These results suggest that many current models struggle to learn transferable substrate–enzyme interaction principles and instead exploit dataset‐specific correlations.

Importantly, these shortcomings do not imply that machine learning is inherently unsuitable for enzyme mining. Rather, they emphasize the importance of rigorous best practices, including careful training data curation, cluster‐aware data splitting, substrate‐held‐out benchmarks, and strict control of information leakage. As highlighted in recent reviews [28, 121], fair and task‐relevant evaluation schemes are essential for estimating real‐world utility and avoiding overly optimistic conclusions about model performance and generalizability.

Training data quality remains a central determinant of model performance and generalizability. Beyond dataset size, key factors include the accuracy and consistency of functional annotations, sufficient coverage of enzyme sequence and chemical space, and transparent reporting of both active and inactive measurements. Too often, inconsistencies in assay conditions, incomplete metadata, or missing information on experimental uncertainty limit the reuse of published data for machine learning. This could be addressed by wide adoption of standardized reporting practices, such as the STRENDA guidelines [123, 124], together with structured, machine‐readable data formats like EnzymeML [125]. This would substantially enhance data quality, facilitate integration across studies, and enable more reliable training and benchmarking of machine learning models for enzyme discovery.

Another persistent limitation across current datasets is strong taxonomic sampling bias, limiting the sampled sequence space: bacterial and cytosolic enzymes dominate training data, largely due to historical focus and the ease of heterologous expression in *E. coli*. Recent high‐throughput screening efforts are expanding coverage into plant‐ and fungal‐specialized metabolism [36, 69], but many biotechnologically important enzyme classes remain underexplored, including unspecific peroxygenases, cytochrome P450s, and enzymes requiring post‐translational modifications. Progress in these areas will require not only targeted experimental data generation but also molecular representations capable of capturing cofactors, post‐translational modifications, and cellular context [126].

Looking ahead, tighter integration between machine learning and experimentation will be essential to unlock the full potential of machine learning‐driven enzyme mining. Closed‐loop workflows that combine Bayesian experimental design with rapid assay technologies offer a promising route toward transforming enzyme discovery from a static prediction task into an adaptive, data‐efficient process. Advances in ultrahigh‐throughput screening, including PUSDA‐based approaches [127] and microfluidic platforms [65, 108], provide the technological foundation for such iterative design–build–test–learn cycles.

In this context, it will be increasingly important to move beyond simple ranking of enzyme candidates based solely on predicted probabilities or turnover numbers. Most current models provide point estimates without conveying uncertainty, despite prediction confidence being strongly influenced by data diversity, noise, and task difficulty. Distinguishing between highly probable yet uncertain predictions and moderately probable but reliable ones would enable better‐informed experimental decision‐making. In parallel, integrating economic and sustainability metrics—such as production cost of the enzyme determined by expression levels and stability under process conditions, substrate availability, and environmental impact—alongside activity and selectivity predictions will better reflect real biocatalytic constraints.

Ultimately, we believe that the impact of machine learning will extend beyond accelerating the discovery of robust and sustainable biocatalysts toward actively guiding experimental decision‐making in the context of the practical realities of industrial biocatalysis.

## Funding

This study was supported by the Nederlandse Organisatie voor Wetenschappelijk Onderzoek (https://doi.org/10.61686/ZXZCQ29832).

## Conflicts of Interest

The authors declare no conflicts of interest.

## Data Availability

Data sharing is not applicable to this article, as no datasets were generated or analyzed during the current study.
